# Depth-to-bedrock map of China at a spatial resolution of 100 meters

**DOI:** 10.1038/s41597-019-0345-6

**Published:** 2020-01-03

**Authors:** Fapeng Yan, Wei Shangguan, Jing Zhang, Bifeng Hu

**Affiliations:** 1 Southern Marine Science and Engineering Guangdong Laboratory (Zhuhai), Guangdong Province Key Laboratory for Climate Change and Natural Disaster Studies, School of Atmospheric Sciences, Sun Yat-sen University, Guangzhou, China; 2 College of Global Change and Earth System Science, Beijing Normal University, Beijing, China; 3 Unité de Recherche en Science du Sol, INRA, Orléans, 45075 France; 4 InfoSol, INRA, US 1106, Orléans, F-4075 France; 5 Sciences de la Terre et de l’Univers, Orléans University, 45067 Orleans, France

**Keywords:** Geology, Hydrology

## Abstract

Depth to bedrock influences or controls many of the Earth’s physical and chemical processes. It plays important roles in soil science, geology, hydrology, land surface processes, civil engineering, and other related fields. However, information about depth to bedrock in China is very deficient, and there is no independent map of depth to bedrock in China currently. This paper describes the materials and methods to produce high-resolution (100 m) depth-to-bedrock maps of China. For different research and application needs, two sets of data are provided for users. One is the prediction by the ensemble of the random forests and gradient boosting tree models, and the other is the prediction and the uncertainty of prediction based on quantile regression forests model. In comparison with depth-to-bedrock maps of China extracted from previous global predictions, our predictions showed higher accuracy and more spatial details. These data sets can provide more accurate information for Earth system research compared with previous depth-to-bedrock maps.

## Abstract (summary)

**Table:** 

Measurement(s)	depth
Technology Type(s)	computational modeling technique
Factor Type(s)	biomass yield • slope • wetness • rock type • soil type • geographic location
Sample Characteristic - Environment	land
Sample Characteristic - Location	China

Machine-accessible metadata file describing the reported data: 10.6084/m9.figshare.11358929

## Background & Summary

Bedrock is the consolidated solid rock underlying unconsolidated surface materials, such as soil or other regolith^1^. Depth to bedrock (DTB) is equivalent to the total thickness of the solum and weathered rocks. DTB information plays an important role in many fields of Earth system science. In soil science, DTB is a key indicator of soil resources, because it restricts root penetration of plants. DTB is a key variable provided by global soil projects such as GlobalSoilMap (http://www.globalsoilmap.net/) and SoilGrids project (http://soilgrids.org). In geology, DBT has been used for applications such as mineral exploration, earthquake modelling, and landslide risk assessment^2,3^. In land surface modelling, DTB is an important input parameter that affects the energy, water, and carbon cycles^4^. DTB information is also indispensable to civil engineering in building homes, roads, railways, and bridges^5^. Furthermore, DTB is of great importance to the study and applications of hydrology, ecology, agriculture, and other relevant fields^6,7^.

Although information about DTB is very important, to date, information about DTB in China is very deficient, and there is no independent map of DTB in China. However, researchers have advanced toward this target. Globally, there are several existing maps of DTB covering the area of China. The earliest global distribution of DTB was produced by the Food and Agriculture Organization (FAO)^8^, but the depth was limited to the uppermost 2 m. This map is produced by assigning a representative sample for each soil type on the FAO soil map with the scale of 1:5,000,000. Hengl *et al*.^9^ developed a global depth-to-bedrock map at 1-km resolution based on zero-inflated models. Pelletier *et al*.^10^ produced a global data set of the average thicknesses of soil, intact regolith, and sedimentary deposits at 1-km resolution by geomorphically based models. Shangguan *et al*.^11^ produced another global map of depth to bedrock based on an ensemble of machine learning (i.e., random forest and Gradient Boosting Tree), using soil profile data, borehole data, and pseudo-observations.

Among the above-mentioned maps of DTB, there are still some deficiencies, including coarse resolution, limited observations, and limited accuracy. Most of them have relatively coarse resolutions (1 km or coarser), except that the map produced by Shangguan *et al*. (2017) is 250 m^11^. However, several environmental covariates (mainly remote sensing data) with high resolution have been produced, which can be used to produce a high-resolution DTB map of China. Though remote sensing can only see the very top surface, the surface or subsurface conditions such as topography and vegetation may affect how deep is the bedrock. For example, high-slope areas (which can be derived from remote sensing DEM data) often have higher water loss and more severe erosion. After long-term development, these areas are more likely to have relatively low DTB. In addition, observations of DTB by the FAO^8^, Shangguan *et al*.^12^ and Hengl *et al*.^9^ have been based solely on soil data; thus, the predictions are often limited to soil surfaces with depths limited to several meters. These depths are not consistent with the actual distribution of DTB. In addition, most samples of Pelletier *et al*.^10^ and Shangguan *et al*.^11^ were located in North America, whereas no samples (Pelletier *et al*.^10^) or only a small number of samples (598 for Shangguan *et al*.^11^) were located in China, which resulted in high uncertainty for predictions in China. However, a large number of borehole logs produced by geologists in China provide DTB information and are now available. Both the site observations of boreholes and environmental covariates provide the cornerstone for producing a new map of DTB with higher accuracy and higher resolution (and thus more spatial details).

Various methods were used as spatial prediction models for mapping, including geostatistical models and machine learning. Geostatistical models were widely recognized as primary spatial prediction techniques from the 1970s^13^ and are still in use^14,15^. Recently, there is an increasing trend of using machine learning methods in spatial predictions, especially in the last ten years^16–18^. In most cases, machine learning techniques have higher performance over simpler approaches (including geostatistical methods) as spatial prediction models due to the comparison in many studies^16,17^. In this study, we used three tree-based machine learning algorithms, i.e., random forest (RF), gradient boosting tree (GBT) and quantile regression forests (QRF), to produce the DTB maps.

**Fig. 1:**
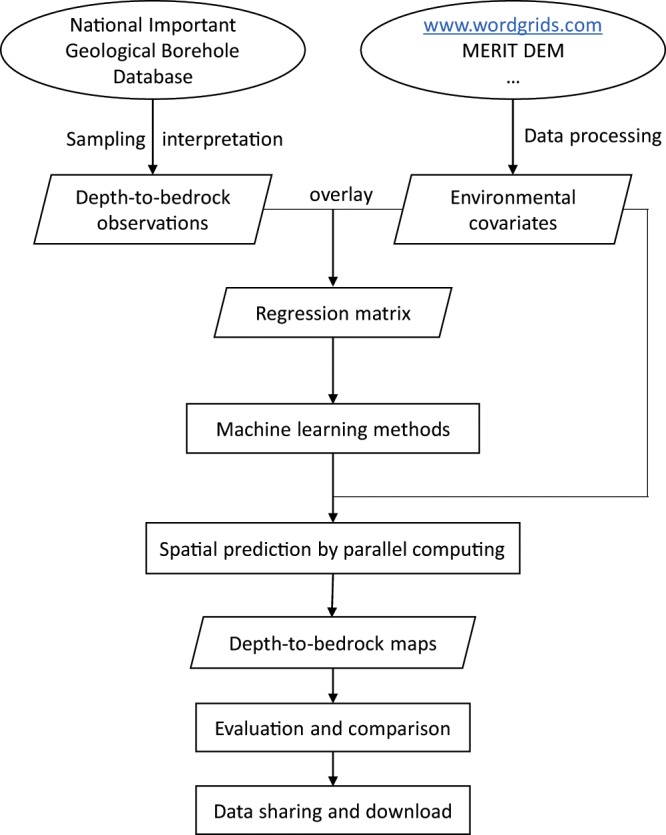
A brief overview of this study.

This study developed DTB maps of China at higher spatial resolution and higher accuracy. These maps will provide more accurate information of DTB in China for Earth system science related research, such as mineral exploration, land surface modelling, hydrology modelling and so on. Figure 1 shows a brief overview of this study.

## Methods

### Establishment of DTB observation datasets

A total of 6,382 DTB observations mainly sampled from the Chinese National Important Geological Borehole Database (NIGBD: http://zkinfo.cgsi.cn) were used in our study. These observations also contain a small part (about 2%) of pseudo-observations of DTB generated from expert knowledge.

#### Observations sampled from the NIGBD

The DTB of every borehole must be interpreted manually and interpreting more than 80 million boreholes logs therefore demands an immense amount of work and has high costs. However, many boreholes that are located close to each other have similar DTB and environmental factors. Therefore, we developed a sampling scheme (named stratified-additive sampling) to take a fraction of the borehole drillings from the NIGBD as the observation data set in this study. This scheme, which is designed to avoid spatial clustering by taking only one observation from each 0.2 × 0.2 arc-degree grid, is described in two parts in the following.

The first part is a sampling scheme similar to stratified sampling. Mapping methods, regardless of methods based on spatial autocorrelation or soil environmental correlation, have requirements based on the number, distribution, and typicality of the samples, which ensure global representation of the samples^19^. To obtain representative samples from these boreholes, we used a sampling scheme similar to stratified sampling to acquire our training points from the NIGBD. The stratified sampling scheme includes designation of grid shape (such as a square grid, triangular grid, or hexagonal grid) and grid size. A square grid is the easiest and most effective, and is most widely used in sampling^19^. In general, smaller grid size leads to more accurate predictions, but with greater sampling costs. Here, we used square grid sampling with a 0.2 × 0.2 arc-degree grid, in consideration of the balance between representativeness and cost. Usually, one observation or a number of observations are sampled at random locations from each grid. However, the locations of boreholes in this study were determined in a previous geological survey. Thus, we have taken one borehole randomly from each grid instead of one borehole from a random location in stratified sampling. In each 0.2° × 0.2° grid, there may be no observation, one observation or multiple observations at different locations. If there is no observation in a grid, it results in vacancy of observation of this grid. If there is one observation, we use it. If there are multiple observations, we take one observation randomly among them. The second part is an additive sampling method. The depths of the boreholes range from 0 m to more than 1 km. Among these boreholes, we were unable to determine the DTB from a few because of the limitations of the records (see details in Sect. 2.1.2). This constraint resulted in vacancies of many grid cells after the interpretation of all boreholes from the first sampling. To resolve this problem, we used an additive sampling method; that is, additional samplings were taken multiple times until no new observations could be added to the sampled data sets. Thus, the latter samplings were aimed at grids without DTB data by interpretation based on the previous samplings. After the first sampling, we obtained one borehole profile for each 0.2° × 0.2° grid to the extent that was possible. Then, we aimed to interpret all the borehole profiles to determine the DTB value, but we were sometimes unable to interpret the DTB. Consequently, some grids still have no DTB observations although these grids had more boreholes other than the borehole in the first sampling. In the second sampling, we tried to sample another borehole for each of these grids without DTB interpretation. Then, we aimed interpret all the new borehole profiles in the second sampling to obtain the DTB values, but we were sometimes still unable to interpret the DTB. A third sampling was then taken and so on. After a finite number of additive samplings, the borehole logs of the NIGBD were considered efficiently used, and samples from all the samplings were used in our study. The spatial distribution and statistics of DTB observations interpreted from boreholes is introduced in Data Records section.

#### Interpretation of borehole records

**Fig. 2:**
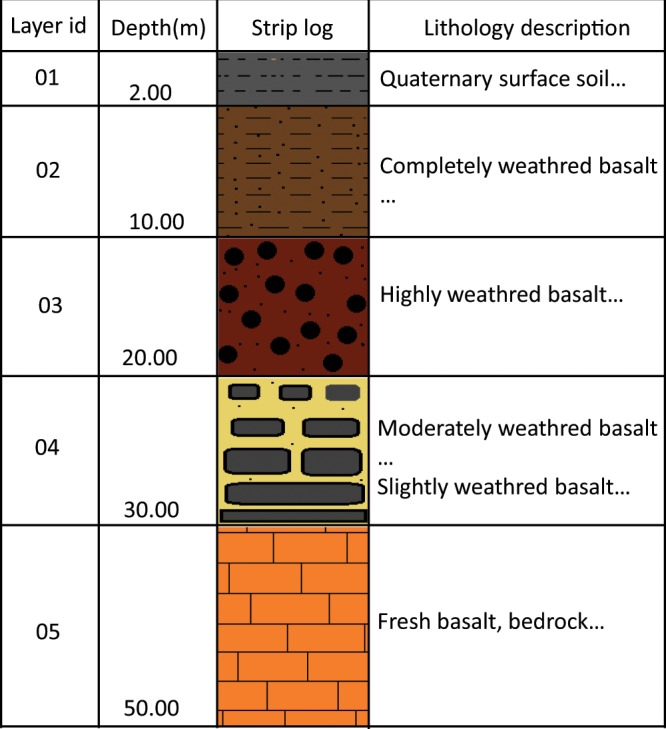
A typical borehole log sketch column. A borehole log describes the materials, color, and composition of each layer, and provides the depth, dip, and other relevant information. The original logs are in Chinese.

Interpreting DTB from borehole profiles sampled from the NIGBD was one of the crucial aspects of this study. Borehole profiles, which were previously recorded by geologists, have longitudinal verbal descriptions of soil layers and lithological layers with corresponding depths from the land surface to the top and bottom of each layer. A typical simplified borehole profile diagram is shown in Fig. 2.

Each borehole profile has several layers. Generally, the top layer of a borehole profile is pedolith, where pedological processes have destroyed the original bedrock structure, principally through the weathering of primary bedrock minerals and the formation and re-distribution of secondary materials. Below is saprolite, referring to the zone where the bedrock fabric is largely isovolumetrically weathered but primary bedrock structures are still recognized. At the bottom is the unweathered bedrock. Because different boreholes were drilled by different geological teams at different times, the details of stratification in the profiles often differ, and the lithological description of each layer may be detailed or vague. These differences result in inconsistencies or uncertainties in the borehole database, which were propagated into our DTB observations. To reduce the error of DTB in the process of interpreting borehole profiles as much as possible, we did not use borehole profiles whose lithological descriptions were too vague (such as a layer that is composed of bedrock mixed with weathered rocks) to accurately determine the DTB.

To interpret the DTB from a borehole profile in the form of a scanned picture, we manually determined the boundary between the regolith and fresh bedrock based on lithological descriptions and the dip angle of the borehole. There are four kinds of cases in the interpretation of DTB. In the first case, DTB is reached for most boreholes and the dip angle is 90°. Then, the DTB is taken as the boundary depth. In the second case, a minority of boreholes have a dip angle less than 90°. Then, the DTB is calculated as the product of the boundary depth and the sine of the dip angle. In the third case, some boreholes are too shallow (several meters or less than 1 m) to reach the bedrock, and some have lithological records that are unclear, which can make it is very difficult to determine the DTB (as described in Sect. 2.1.1). Therefore, we used additive samplings to select another borehole from the grid. In the fourth case, because a number of boreholes were drilled to depths of more than 100 m but still did not reach the bedrock, we could not obtain accurate DTB data from the borehole profiles. In this case, we regarded the depths of those boreholes as approximations of the real DTB value as most research and applications focus on relatively shallow depths.

#### Pseudo-observations

As shown in Fig. 1, DTB observations interpreted from borehole logs cover an extensive area across China, except for the Qinghai-Tibet Plateau where boreholes are difficult to drill. Any purely data-driven model fitted with large gaps in the covariate space is most likely to result in considerable omissions, especially for areas that are often inaccessible or not of interest for soil surveys or geological exploration. Therefore, we used pseudo-observations added to training data to fill such gaps, which will avoid extrapolation for these areas (e.g., deserts and steep mountainous areas). Deserts consist mainly of sand, and the DTB of such areas could be found in some publications. Steep-slope areas without vegetation typically have very shallow or zero DTB; that is, rock outcrop. Therefore, we used the following data sources to generate pseudo-observations to add to the training points:

1. The distribution map of deserts in China from the Data Center of Environmental and Ecological Science in Western China (http://zgsm.westgis.ac.cn).
2. Steep, bare surface areas generated using a slope map of China and remote-sensing-based data.
3. Previously published detailed geological maps reporting DTB or bedrock outcrops.

We generated a certain number of points in random positions within deserts based on the distribution map of China’s deserts. The DTB values of these points were obtained from existing material and previous studies of the sand thickness of the deserts. We obtained some information about the thickness of desert and sand dunes from websites such as Baidu Encyclopedia (https://baike.baidu.com). Some publications provide information on the profiles of China’s deserts, from which we obtained about 40 pseudo-observations. Boreholes logs from Pishan, Moyu, and Yutian in the Taklimakan Desert show that the subsurface of this desert is mainly medium to fine-grained sand and silt with a thickness less than 200 meters^20^. The Shashan zone at the south rim of the Taklimakan Desert has a thickness of less than 80 m^21^. Areas where the slope is greater than 60°were extracted from a slope map of China. Then, we randomly generated about 100 points and their DTB values were set as 0.01 m. The number of points was limited to less than 2% of the total number of observations to avoid adding too many soft observations, and we only used points whose values had high credibility based on the information source.

### Preparation of environmental covariates

In our study, a total of 147 related environmental layers, which cover five types of factors (climate, topography, living organisms, water dynamics, and parent material) and represent the factors of soil formation according to Jenny^22^, were selected to generate a DTB map of China. Although DTB is somewhat different from basic soil properties, it is closely related to soil because bedrock is a kind of parent material for soil. Soil is developed based on bedrock or regolith such as deposit via physical, chemical and biological processes. Factors affecting soil development are also related DTB. Therefore, it is reasonable to include factors of soil formation in the prediction of DTB. However, DTB is also influenced by geological characteristics and processes such as rivers, glaciers, fractures and partings in the rock, moraine, geological age, erosion, deposition, periglacial processes and so on^23–25^. So in this study, the conceptual framework is DTB = f (s, c, o, r, g, a, n) improved from S = f (s, c, o, r, p, a, n) proposed by McBratney *et al*.^26^, where “g” stands for geological factors. The 147 covariates classified as “scorgan” factors included:

1. Harmonized soil database images: percent coverage of Andosols, Histosols, and dozens of other soil types.
2. Climatic images: images indicating the values of 8-day MODIS day-time and night-time local standard time (LST), long-term and monthly precipitation data, etc.
3. Land use and land cover images: including vegetation maps, land cover and land use classifications, biomass and yield maps, etc.
4. Relief data, mainly derived from digital elevation models: slope maps, the topographic wetness index, the topographic openness index, physiographic landform units, elevation and secondary terrain attributes, etc. These covariates were calculated by R and SAGA-GIS.
5. Geological and parent material maps: rock type based on the global lithology and geological ages based on surface geology.

A complete list of the 147 environmental covariates is given in the covariate list file^27^. Covariates whose resolution are not 100 m are processed into images in 100 m via geographic information system when generating predictions. There are three situations where we treat these covariates: (1) for images with a spatial resolution coarser than 100 m, we resampled them into images with a resolution of 100 m with identical values for the finer cells within an original coarse cell; (2) for attribute images with a resolution finer than 100 m, we aggregate them to images with a resolution of 100 m by averaging; (3) for classification images with a resolution finer than 100 m, we aggregate them to images with a resolution of 100 m by assigning the majority class.

### Spatial prediction model

**Fig. 3:**
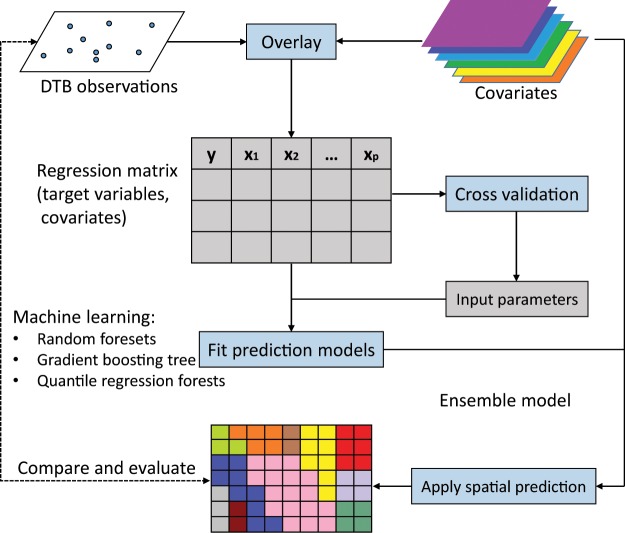
The spatial prediction framework used to fit models and apply spatial prediction of DTB in China at 100 m resolution.

The framework of our research is shown in Fig. 3, which is based on the work of Hengl *et al*.^28^ and Shangguan *et al*.^11^. This framework consists of four main processes:

1. Overlaying observations of DTB and covariates to generate a regression matrix for modelling;
2. Obtaining the best parameters for modeling using cross-validation;
3. Fitting the prediction models based on the whole regression matrix;
4. Applying spatial prediction models using covariates and comparing the prediction with existing maps;

#### Model fitting

In this study, we overlaid observations of DTB and covariates under the same coordinate reference to generate a matrix including DTB and covariate columns. The matrix was used as input data for machine learning. Then, we separately used RF and GBT to fit the prediction models. Finally, the spatial predictions were generated using an ensemble model based on the two models. RF and GBT are decision-tree-based ensemble methods whose predictive accuracy are not influenced by collinearity between variables^29^. The RF model uses fully grown decision trees and reduces error by reducing variance^30^. The parameters after optimaliztion in RF model are: mtry = 18, ntree = 1000 and nodesize = 5. The GBT model uses shallow trees and reduces error mainly by reducing bias, and to some extent by reducing variance by aggregating the outputs from many models^31^. We used an ensemble of the two models because ensemble predictions of strong learning algorithms have been shown to be more effective in producing better results^32^ and avoid the overshooting effect^33^. RF and GBT were implemented respectively in the “randomForest” and “xgboost” packages in the R environment. Parallel computing was employed to improve data processing efficiency.

#### Model validation and evaluation

Ten-fold cross-validation was used to evaluate prediction accuracy. Comparison with previously existing DTB maps was then employed to evaluate our results. In cross validation, samples were divided into a training set (5,740 samples) and validation set (642 samples). The training set was used to fit the models, and the validation set was used to validate model performance. Some widely used indicators such as the coefficient of determination (R^2^ or the amount of variation explained by the model), mean error (ME), relative error (RE), and root mean square error (RMSE) were used to evaluate model performance. Of these indicators, the coefficient of determination is calculated by:

1 $${{\rm{R}}}^{2}=\frac{SSR}{SST}=1-\frac{SSE}{SST}=1-\frac{\mathop{\sum }\limits_{{\rm{i}}=1}^{{\rm{n}}}{({{\rm{y}}}_{{\rm{i}}}-{\widehat{{\rm{y}}}}_{{\rm{i}}})}^{2}}{\mathop{\sum }\limits_{{\rm{i}}=1}^{{\rm{n}}}{({{\rm{y}}}_{{\rm{i}}}-\bar{{\rm{y}}})}^{2}},$$

where SSR is the regression sum of squares, SST is the total variation sum of squares, and SSE is the residual sum of squares, which is the difference between SST and SSR. The variable *y*_*i*_ is the measured target value, ${\hat{\rm y}}$ is the prediction of each point, ${\bar{\rm y}}$ is the average of the measurements, and *n* is a number of validation points. The value of *R*^*2*^ is usually between 0 and 1; a value close to 1 indicates a perfect model, and values near 0 indicate a failed model. The RMSE, which is also called standard error, is calculated by:

2 $${\rm{RMSE}}=\sqrt{MSE}=\sqrt{SSE/n},$$

where MSE is the mean squared error. RMSE estimates the deviation between predictions and observed values. A smaller RMSE indicates a better prediction.

We used the feature selection of RF and GBT to remove some unimportant covariates and avoided the collinearity of variables. We first used all the covariates to fit a model, and then some covariates with low importance (including covariates with high collinearity) were dropped in the final model. Covariates with no or weak relations with DTB may produce noise in fitted models. This noise results in higher errors of predictions. Our results based on modeling with different covariates showed that the noise had a certain degree of influence on the accuracy of the models, especially for the GBT model. In addition, some of the covariates may have data quality and consistency problem, which would introduce error to the prediction. Therefore, we removed some covariates with low importance based on the RF and GBT to reduce prediction errors, model complexity and computation time (this is called feature selection in machine learning). Because there are some limitations of the importance and the importance of correlated covariates is underestimated^34^, a covariate was removed only when it did not make the model without this covariate significantly worse; that is, when the R^2^ of the model decreased less than 0.01 or increased. In this way, we kept a balance between model complexity (i.e., number of covariates) and model accuracy. In addition, to verify whether our predictions were more accurate than existing DTB maps of China, we compared our predictions with existing DTB maps using the validation set.

#### Model prediction and uncertainty estimation

The final model was fitted based on all samples with parameters selected by cross-validation. The final spatial predictions were generated using an ensemble model based on RF and the GBT method. To predict DTB in China at 100-m resolution, we used the available environmental covariates at 100 m resolution.

Because any model for digital soil mapping inevitably suffers from different sources of error^18^, it is important to quantify the uncertainty associated with the produced maps^35^. Analysis and evaluation of uncertainty help data users to understand its existence and can also help to improve decision quality^36^. In this study, we used quantile regression forests (QRF) to estimate the uncertainty of estimations. QRF is a tree-based ensemble algorithm for estimation of conditional quantiles. This method is particularly suitable for high-dimensional data. QRF were implemented via the R environment in the “*quantregForest*” package^37^. We used the same 53 covariates of the final RF model for quantile predictions of the QRF model. To estimate the uncertainty of predictions at every location, we generated the uncertainty map of predictions by:

3 $$uncertainty=\frac{q{p}_{0.9}-q{p}_{0.1}}{q{p}_{0.5}},$$

where *qp*_0.9_ is the 0.9 quantile prediction of DTB, *qp*_0.1_ is the 0.1 quantile prediction of DTB, and *qp*_0.5_ is the 0.5 quantile prediction of DTB.

For different research and application needs, two sets of data are provided for users. One is the prediction by the ensemble of the RF and GBT models, and the other is the prediction and the uncertainty based on QRF. Because most users do not need an uncertainty map in their applications, it is recommended to use the ensemble prediction because it is more robust. In cases where consistent prediction and uncertainty are needed, it is recommended to use the estimation by QRF.

#### Parallel computing in spatial prediction

In order to avoid the short of memory caused by high-resolution mapping and improve computational efficiency of spatial prediction. We used parallel computing to apply spatial prediction.

Firstly, we divided all the covariates into several 1° × 1° block. Based on the final model, covariates in 1° × 1° block were used to apply spatial prediction in current block computed by a core. Multicore computing can process multiple block in the same time, thus greatly improving the speed of spatial prediction. After obtaining the results of all blocks, we used image mosaic to generate the final DTB map of China.

## Data Records

### Model input

#### DTB Observations

DTB observations are in “.csv” format and named “DTB_observations.csv”. This file is available on Figshare^27^. Every observation (a row) contains longitudinal and latitudinal coordinates and DTB value.

**Fig. 4:**
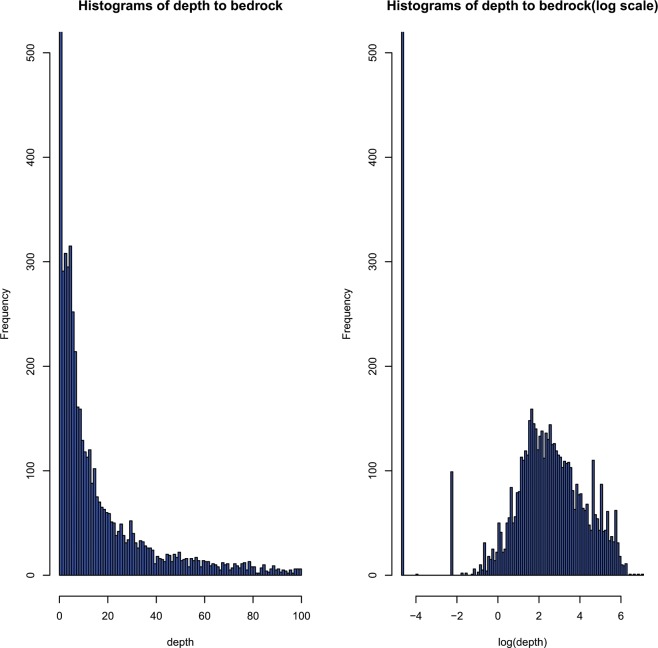
Histogram of depth to bedrock. (**a**,**b**) Distribution of original data and after logarithmic transformation (values large than 100 m are not shown).

**Fig. 5:**
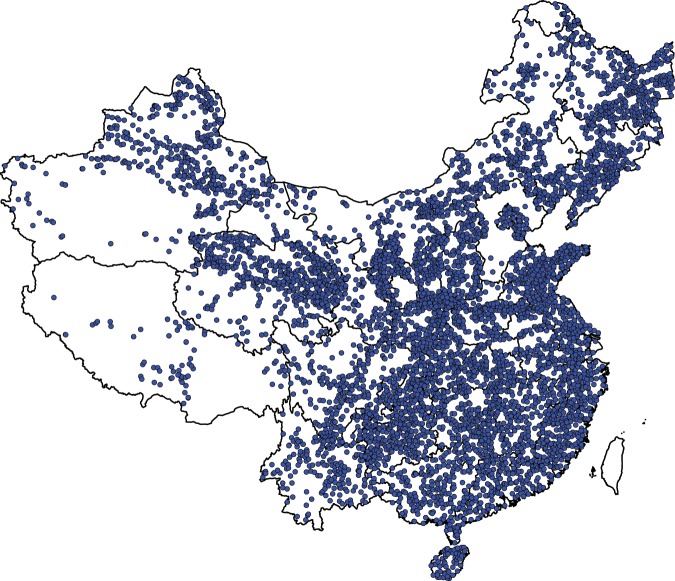
Distribution of DTB observations interpreted from boreholes.

A summary of the DTB statistics is provided in Table 1. The DTB ranged from 0 to 1,106.91 m, with a mean DTB of 36.62 m and a median value of 8.24 m. Figure 4(a) shows a histogram of DTB within 100 m. The DTB after logarithmic transformation had a distribution similar to a normal distribution but with many zero values (i.e., outcrops) (Fig. 4(b)), which is called zero-inflated distribution. The distribution of DTB observations interpreted from boreholes is shown in Fig. 5.

**Table 1:** Summary statistics of depth to bedrock in meters.

DTB	Number	Statistics	Value
=0	1026	Min	0
0~2.00	585	Max	1106.9
2.00~10.00	1833	Median	8.2
10.00~50.00	1768	Mean	36.1
50.00~100.00	427	Variance	5189
100.00~300.00	630		
>300.00	113

#### Covariates

The covariates we used are listed in the covariate list file (“.xlsx” format, named “covariates_list_for_generating_DTB100_of_China.xlsx”)^27^. The data sources of these covariates are provided in this file. Topographic covariates are obtained based on digital elevation model via R and SAGA (see file “DEM_functions.R” on Github: https://github.com/yanfp/DTB100China). The essential information about every covariate are provided in columns. The last three columns show the choice of covariates for the RF, GBT and QRF models, where a value of one indicates that the corresponding covariate was used in the final model. The final RF, GBT and QRF model used 53, 44 and 53 covariates, respectively, which are marked in the covariate list file.

### Prediction results

The resulting maps are available on Figshare^27^ and at http://globalchange.bnu.edu.cn/research/cdtb.jsp. All maps are in “.tif” format. Two sets of maps are provided: DTB map by ensemble model, DTB map and the corresponding uncertainty map by QRF model. These maps were divided into two block by the longitude line of 105°E due to the limitation of data capacity on Figshare. Block1 covers the area from the westernmost to 105°E, and Block2 covers from 105°E to the easternmost.

**Fig. 6:**
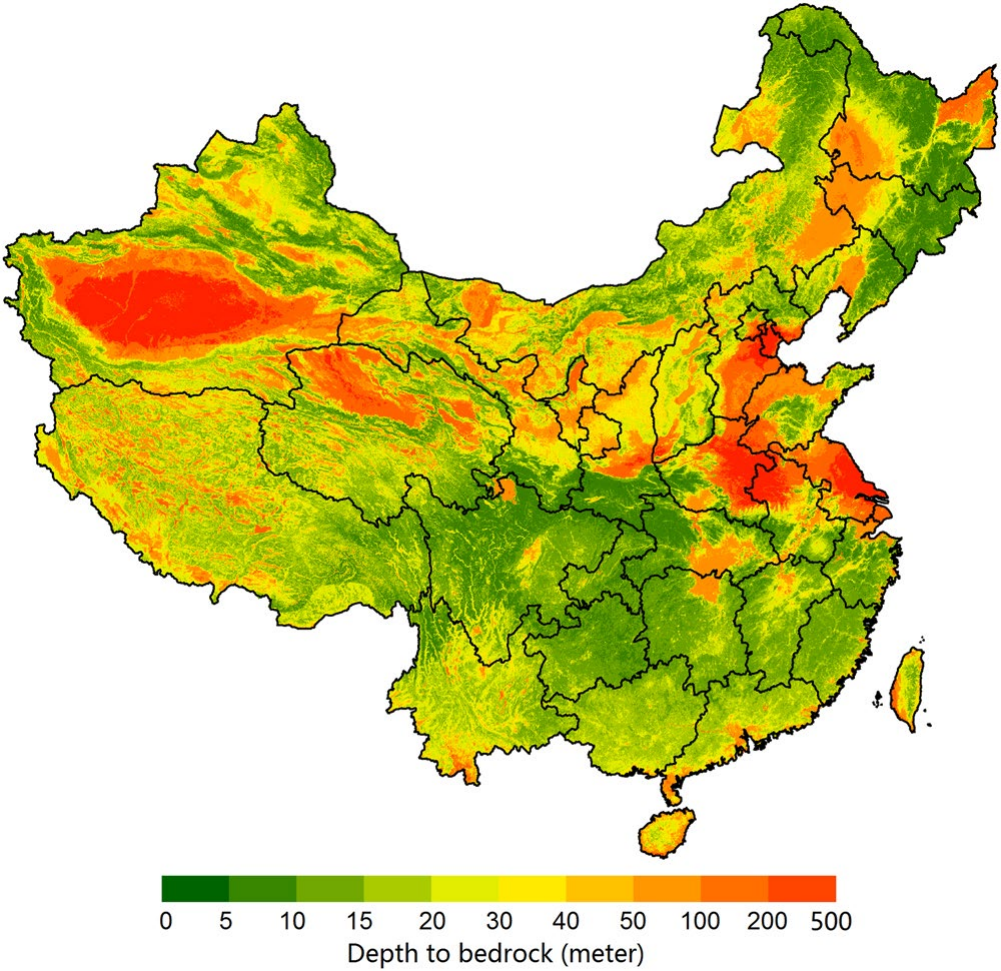
Final prediction of depth to bedrock based on the ensemble model.

Output estimations of DTB by the ensemble model based on RF and GBT at 100-m resolution are shown in Fig. 6. Our estimated results reveal that the predicted mean DTB was 42.20 m. High values of DTB were mainly distributed in desert areas, the North China Plain (including areas in Hebei province, Henan province, and Jiangsu province) and the Northeast China Plain (including areas in Heilongjiang province, Jilin province, and Liaoning province). Relatively lower values of DTB were mainly located in hilly and mountainous areas, such as Sichuan province, Chongqing city, Guangxi province, and the mountainous areas of Northeast China. The spatial pattern of the DTB map of this study is similar to those of the maps produced by Pelletier *et al*.^10^ and Shangguan *et al*.^11^.

**Fig. 7:**
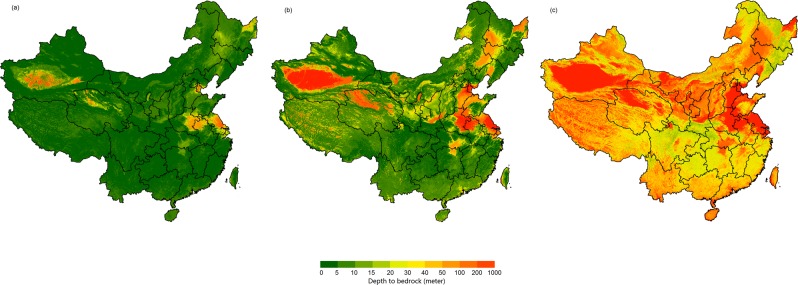
Depth to bedrock maps produced by the quantile regression forests model at three percentiles. (**a**–**c**) The percentiles are 0.1, 0.5, and 0.9.

In addition to predictions by ensemble model, another dataset including median predictions and uncertainty was produced. Estimations of three percentiles (0.1 (Fig. 7(a)), 0.50 (Fig. 7(b), and 0.9 (Fig. 7(c)) were produced by the QRF model. The mean values of the estimated DTB for the three percentiles were 3.05 m, 29.16 m, and 98.14 m, respectively. The maps (Figs. 6 and 7(b)) show that the spatial pattern of DTB predicted by the QRF model was similar to that of the ensemble model based on the RF and GBT methods.

**Fig. 8:**
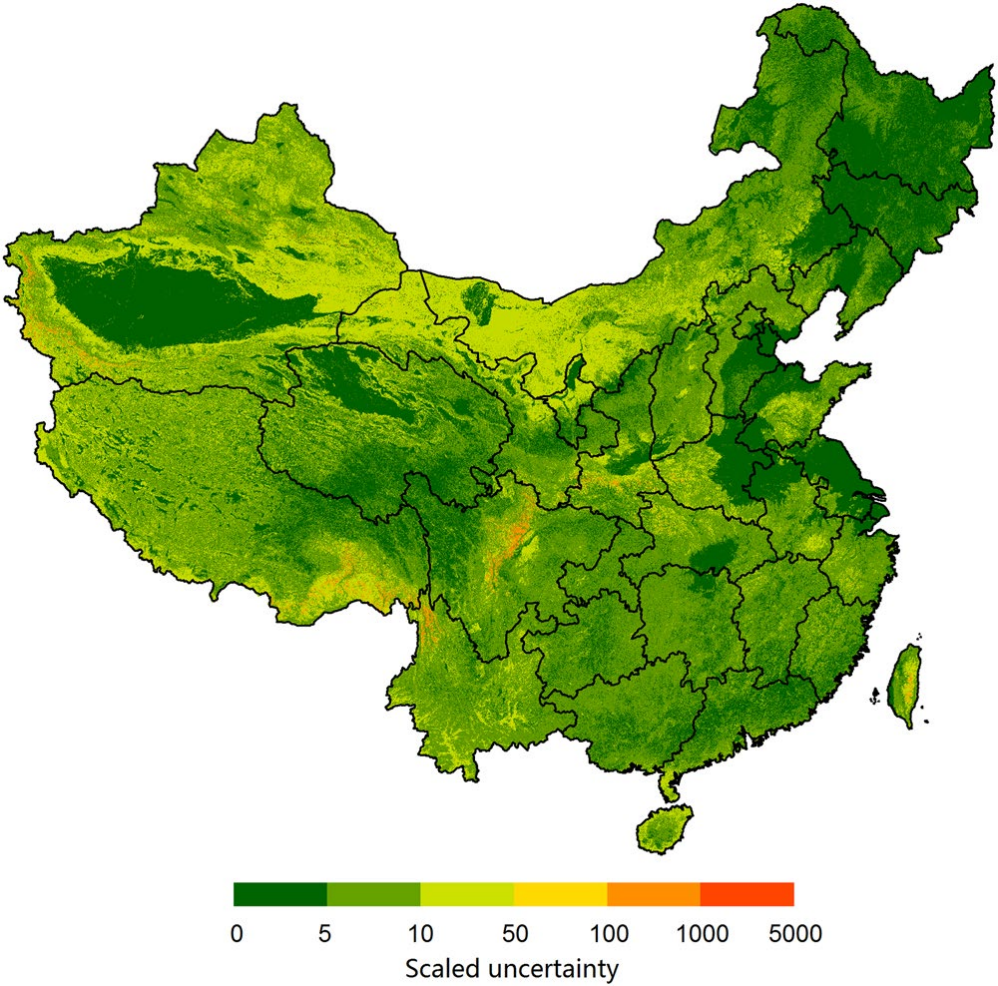
Uncertainty map of prediction of the depth to bedrock.

The uncertainty map of the prediction of DTB is shown in Fig. 8. The uncertainty in the predictions in part depends on the density of sampling^38^. In our study, it was low in deserts, sandy areas, the North China Plain, and the Northeast China Plain, where the topography is relatively simple and sampling was relatively dense. In the Tibetan Plateau and western Inner Mongolia, where sampling was sparse and DTB is low, the uncertainty was high. The uncertainty was also relatively high in the Yun-Gui Plateau where the topography is complex with widespread karst landforms.

## Technical Validation

### Estimation accuracy

**Fig. 9:**
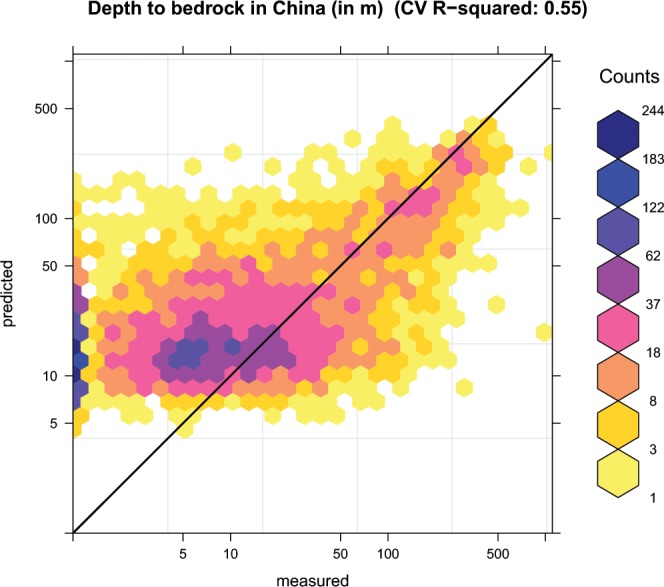
Plot showing cross-validation results for depth to bedrock on a logarithmic scale. *R*^2^ is calculated using Eq. (1).

The cross-validation summary statistics of interpolation for the models based on RF, GBT and QRF are shown in Table 2 and Fig. 9. These statistics show that RF produced more accurate estimations than GBT. Our results showed significant overestimation in lower values of DTB, which is a common problem in regression, especially when the model is not able to explain >50% of the variability in the target variable^11^. In addition, this overestimation may be related to the zero-inflated distribution of DTB. Hengl *et al*.^28^ reported this overestimation for other three zero-inflated observation data including soil organic carbon, bulk density and coarse fragments. It should be noted that spatial clustering of observation is obvious in Fig. 1. The random selection of cross validation gives more importance to the locations with dense samples than those with sparse samples, especially the west part of China. As a result, there is a systematic bias in the way the evaluation is performed.

**Table 2:** Mapping performance for the depth to bedrock.

Model	R^2^	RMSE	ME	RE
Random forests	0.538	49.86	2.12	0.06
Gradient boosting tree	0.537	49.84	0.76	0.02
Ensemble	0.547	49.33	0.68	0.02
Quantile regression forests	0.519	51.15	11.77	0.33

R^2^ is the coefficient of determination, root mean square error (RMSE), ME is mean error, and RE is relative error.

We have also investigated whether using kriging of residuals can improve predictions of DTB. There is no significant spatial autocorrelation structure for residuals, because machine learning models has explained the majority of spatial variation. Kriging of residuals for DTB of China does not seem to be necessary nor is it practical to implement for billions of pixels: it would only marginally improve the accuracy of predictions at high computing costs.

### Comparison with other maps

**Fig. 10:**
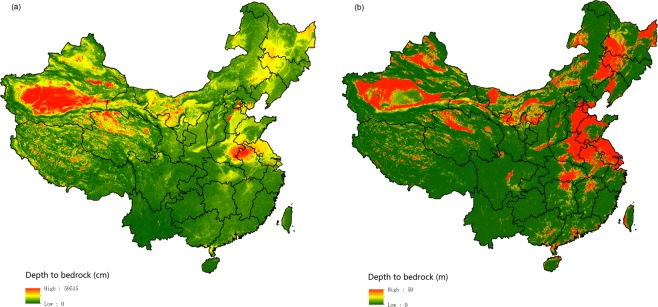
Extracted maps from global predictions by previous studies. (**a**,**b**) Maps by Shangguan *et al*.^11^ and Pelletier *et al*.^10^.

We compared our results with existing maps produced by Pelletier *et al*.^10^ (Fig. 10(a)) and Shangguan *et al*.^11^ (Fig. 10(b)). Our results show similar spatial patterns to these maps. DTB values in deserts, sandy areas, and the North China Plain were relatively high, and values in hilly and mountainous areas, such as Chongqing City and Yunnan province, were relatively low in the map of this study and in maps from global predictions. The estimated mean DTB was 42.20 m in our study, whereas the mean values predicted by Pelletier *et al*.^10^ (Fig. 10(a)) and Shangguan *et al*.^11^ (Fig. 10(b)) were 11.81 m and 26.64 m. Table 3 shows statistics between the 6,328 observations used in this study and the predictions of the three studies. For our map, we used 10-cross-validation to calculate the statistics (note that the RMSE and ME of our study are the same as in Table 2). For the maps by Pelletier *et al*.^10^ and Shangguan *et al*.^11^, we calculated the statistics between the 6,328 observations and the prediction directly, i.e., we used all the observations for the validation. This validation may not be completely fare. But it is still a pragmatic and good way for the comparison. The correlation coefficients between DTB observations and predictions by the ensemble model and QRF model in our study are 0.739 and 0.720 respectively, which are significantly higher than the estimation results of Pelletier *et al*.^10^ and Shangguan *et al*.^11^. In addition, compared with the prediction results of Pelletier *et al*.^10^ and Shangguan *et al*.^11^, our estimation results had obviously lower RMSE and ME.

**Table 3:** Statistics between observations and predictions of three studies.

Study	r*	RMSE	ME	RE
Ensemble model in this study	0.739	49.86	0.68	0.02
QRF model in the study	0.720	51.15	11.77	0.33
Pellertier *et al*.^10^	0.486	81.98	36.52	1.01
Shangguan *et al*.^11^	0.475	67.32	14.71	0.41

**Fig. 11:**
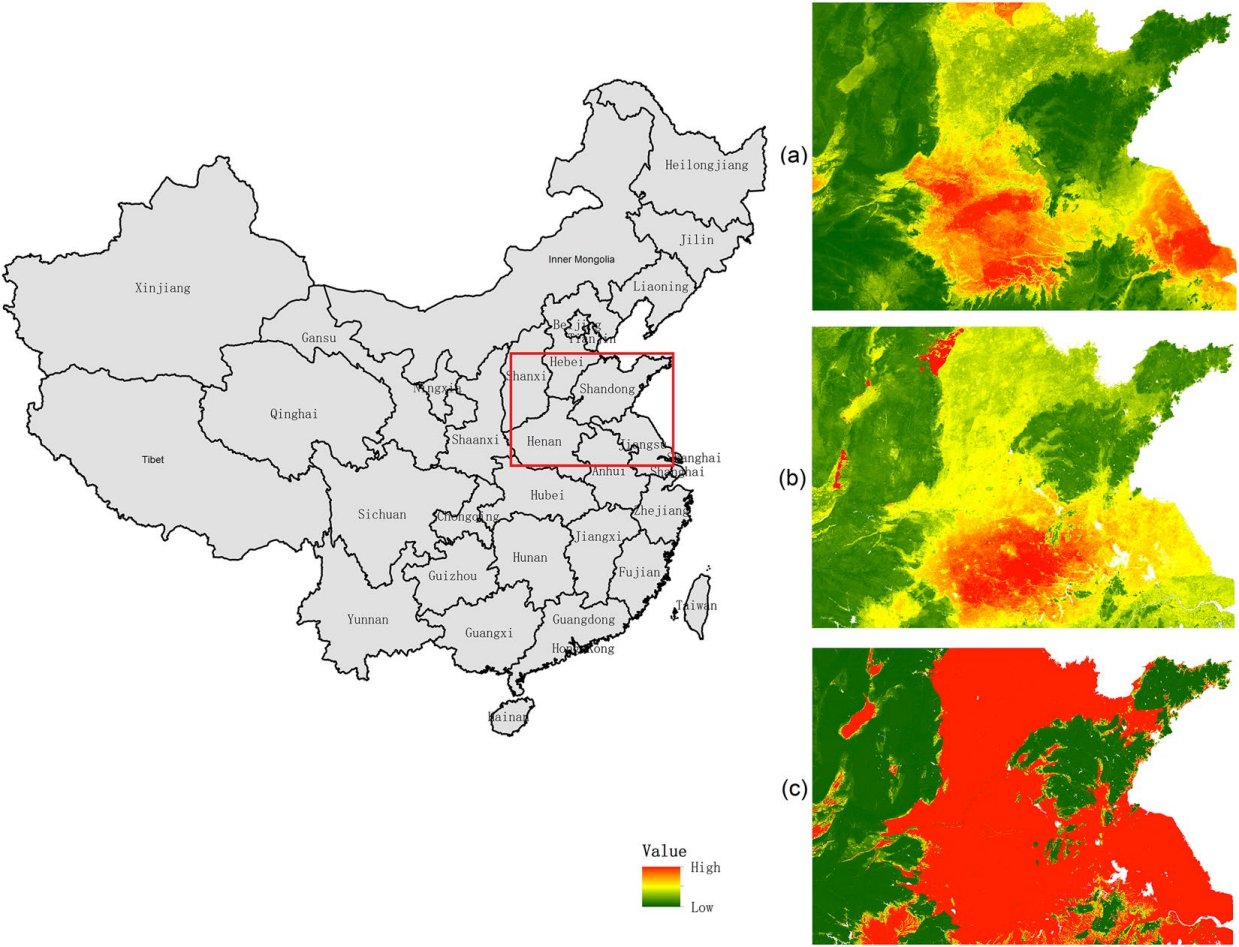
Regional maps of three studies. (**a**–**c**) This study, Shangguan *et al*.^11^, and Pelletier *et al*.^10^.

In addition, our prediction results show similar spatial patterns to the maps produced by Pelletier *et al*.^10^ and Shangguan *et al*.^11^ but revealed more detailed information than previous predictions. There are more jumping points in the map of Shangguan *et al*.^11^ than the others, and the map predicted by Pelletier *et al*.^10^ shows low continuity in space with high values and low values in a wide range. From comparison in a typical region in the North China Plain (Fig. 11), our map revealed more spatial details, especially in high DTB areas, than did the maps by Shangguan *et al*.^11^ and Pelletier *et al*.^10^ (Fig. 11(a)). In contrast, the map estimated by Pelletier *et al*.^10^ shows abrupt change between highland and lowland areas (Fig. 11(c)).

## Data Availability

All code used to generate the predictions is available from the Github (https://github.com/yanfp/DTB100China). The map products were calculated using R version 3.4.1 and packages “randomForest (4.6–14)”, “xgboost (0.71.2)”, “quantregForest (1.3–7)”, “sp (1.3–1)”, “rgdal (1.2–16)”, “raster (2.7–15)”, “pkgmaker (0.27)”, “lattice (0.20–35)”, “plotKML (0.5–8)”, “hexbin (1.27.2)” and “ggplot (3.1.0)”.
